# Supplementary material for: Use of administrative data for evaluating trends in medically-attended Lyme disease, Manitoba, Canada, 2010–2021
Source: PLoS One. 2026 Apr 13;21(4):e0342260. doi: 10.1371/journal.pone.0342260 (PMC13075671; doi:10.1371/journal.pone.0342260)
Supplement: S1 File — (DOCX) [file pone.0342260.s001.docx]

**SUPPLEMENT________________________________________________________________**

**S1 Table. Manitoba Population Research Data Repository (MPRDR) Database**

| Database or Code | Description |
| --- | --- |
| Cadham Provincial Laboratory (CPL) - Laboratory Information Management System (LIMS)^1^ | The central public health microbiology reference laboratory for Manitoba and the sole centre for laboratory services and infectious diseases serology which serves patients, practitioners and public health units in Manitoba. |
| Discharge Abstract Data (DAD)^1^ | Hospital forms/computerized records containing summaries of demographic and clinical information (e.g., gender, postal code, diagnoses, and procedure codes), completed at the point of discharge from the hospital. |
| Drug Identification Number (DIN)^2^ | A computer-generated eight-digit number assigned by Health Canada to a drug product prior to being marketed in Canada. It uniquely identifies all drug products sold in a dosage form in Canada and is located on the label of prescription and over-the-counter drug products that have been evaluated and authorized for sale in Canada. |
| Drug Program Information Network (DPIN)^1^ | Prescription drug claims from all pharmacies for all Manitoba residents, including Registered First Nations, regardless of insurance coverage or final payer. Excludes hospitals, wards, cancer care. |
| International Classification of Diseases, 9th Revision, Clinical Modification (ICD-9-CM)^3^ | Unique 5-digit codes that are assigned allow for accurate billing of services in the outpatient and inpatient clinical setting, data collection, and inventory, and are used by national databases. |
| International Statistical Classification of Diseases and Related Health Problems,  Tenth Revision, Canada (ICD-10-CA)^3^ | Classiﬁes diseases, injuries and causes of death, as well as external causes of injury and poisoning. It also includes conditions and situations that are not diseases but represent risk factors to health, such as occupational and environmental factors, and lifestyle and psychosocial circumstances. |
| Logical Observation Identifiers Names and Codes (LOINC®)^3^ | Clinical terminology used for laboratory test orders and results; international standard for the electronic exchange of clinical health information. |
| Long Term Care Utilization^3^ | Long Term Care Utilization data is maintained by Manitoba Health and consists of records of chronic and rehabilitative services provided by long term care institutions in Manitoba, including hospital patients awaiting placement. |
| Manitoba Health Insurance Registry (MHIR)^1^ | Registry of all individuals registered to receive health services in Manitoba. |
| Medical Claims - Medical Services (MC-MS)^1^ | Claims for visits to physicians/primary care providers in offices, hospitals, and outpatient departments; fee-for-service components for tests such as lab tests. |
| National Ambulatory Care Reporting System (NACRS)^1^ | Hospital-based and community-based ambulatory care at a national level: day surgery, outpatient clinics, and emergency departments. |
| National Rehabilitation Reporting System (NRS)^1^ | Demographic, administrative, and clinical information pertaining to physical rehabilitation. |

1. Manitoba Population Research Repository: [Manitoba Population Research Data Repository Data List | MCHP Concept Dictionary and Glossary for Population-Based Research | Max Rady College of Medicine | University of Manitoba (umanitoba.ca)](http://mchp-appserv.cpe.umanitoba.ca/dataList.php);
2. Government of Canada, Drug Identification Numbers: [Drug Identification Number (DIN) - Canada.ca](https://www.canada.ca/en/health-canada/services/drugs-health-products/drug-products/fact-sheets/drug-identification-number.html)
3. Canadian Institute for Health Information. Pan-Canadian Primary Health Care EMR Minimum Data Set for Performance Measurement, Version 1.1. Ottawa, ON: CIHI; 2022.: [Pan-Canadian Primary Health Care EMR Minimum Data Set, Version 1.1 (2022) (cihi.ca)](https://secure.cihi.ca/free_products/phc-emr-mds-v1.1-en.pdf)

S2 Table. Operational Definitions for Lyme Disease Algorithms

| 1. Primary Case Algorithm – Emergency Room Visits: |
| --- |
| - Having ≥1 Lyme disease ICD, Tenth Revision, Canada (ICD-10-CA) diagnosis code* at any position in ED/hospital; AND - Having prescription claim of ≥7 days of dispensed antibiotics* (e.g., prescription claim of doxycycline dispensed on 1 January 2012 for a duration of 7 days); AND - At least one course of antibiotics with a dispensing date within 30 days of the diagnosis code. |
| 1. Primary Case Algorithm – Hospitalized Cases: |
| - Having ≥1 LD ICD, Tenth Revision, Canada (ICD-10-CA) diagnosis code* at any position in ED/hospital. |
| 1. Primary Care-Adapted Algorithm: |
| - Having prescription claim of ≥7 days of dispensed antibiotics*; AND - Having ≥1 relevant LD ICD-9 code* in Manitoba primary care setting; AND - At least one course of antibiotics with a dispensing date within 30 days of the diagnosis code |
| 1. Serology-Based Algorithm: |
| - Having a positive serology test result^†^ by western blot assay** |
| **The earliest date of diagnosis code or antibiotic dispensing date occurring within 30 days of diagnosis code was defined as the index date.*  *** The date of specimen collection is defined as the index date*  *† A standard two-tiered algorithm comprised of first testing a specimen for total antibodies, followed by further testing for specific antibodies using western blot.* |

**S3 Table. Coding Algorithm Used to Identify Lyme Disease Cases**

|  | ICD-10-CA | ICD-9-CM | DIN | LOINC |
| --- | --- | --- | --- | --- |
| LD | A69.2, M01.2* | 08881 |  |  |
| Antibiotics |  |  | **See Table S6** |  |
| Laboratory test |  |  |  | **See Table S5** |

**S4 Table. Laboratory Codes for Lyme Disease Diagnostic Assays**

| Assay | LOINC |
| --- | --- |
| VlsE1/pepC10 Borrelia (Lyme) IgM/IgG Enzyme-linked immunoassay (ELISA) | 100711-1, 16478-0, 16480-6, 20449-5, 31155-5, 38173-1, 40612-4, 43842-4, 44455-4, 46248-1, 5060-9, 5062-5, 5064-1, 51742-5, 51743-3, 51744-1, 51747-4, 83081-0, 98205-8 |
| Western blot assay (IgG/IgM) | 12781-1, 12873-6, 12874-4, 12877-7, 12878-5, 12879-3, 12890-0, 12891-8, 12892-6, 12896-7, 13502-0, 13503-8, 18201-4, 18203-0, 21116-9  21117-7, 27982-8, 27985-1, 27986-9, 28002-4, 29898-4, 32666-0, 42238-6, 44452-1, 44946-2, 44947-0, 44948-8, 44949-6, 49977-2, 49979-8, 49981-4, 49983-0, 49992-1, 49994-7, 49996-2, 49997-0, 51745-8, 51746-6, 60342-3, 60343-1, 62342-1, 6320-6, 6321-4, 9587-7, 9588-5  9589-3, 9590-1, 9591-9, 9592-7, 9593-5, 9594-3, 9595-0, 9596-8, 9597-6, 9598-4, 9599-2, 96429-6, 96430-4, 96431-2, 96432-0, 98204-1, 98206-6, 94476-9, 94477-7 |

**S5 Table. Drug Identification Numbers for Antibiotic Treatment**

| Antibiotic | Drug Identification Number (DIN) |
| --- | --- |
| Doxycycline | 740713, 874256, 887064, 2375885, 860751, 2512645, 2351234, 2351242, 725250, 2158574, 2247104 |
| Amoxicillin | 2477726, 2237154, 2352710, 2352729, 2352753, 2352788, 2401509, 2401541, 2434709, 2434717, 2241826, 2241827, 2525348, 2525356, 2243224, 2243225, 2514648, 2514656, 2514664, 2514672, 2388073, 2388081, 2458586, 2458594, 2230615, 2230616, 2230617, 2230618, 2433060, 2433079, 2230244, 2532042, 2532050, 2495856, 2495864, 406716, 406724, 452130, 628115, 628123, 628131, 628158, 644315, 644331, 1934163, 2036355, 2492210, 2493381, 2535793, 2535815 |
| Cefuroxime axetil | 2244393, 2244394, 2344823, 2344831, 2212307 |
| Azithromycin | 2480700, 2415542, 2482363, 2482371, 2330881, 2442434, 2523825, 2524449, 2483890, 2465604, 2452308, 2502038, 2479680, 2261634, 2261642, 2310600, 2275309, 2265826, 2332388, 2332396, 2267845, 2212021, 2223724, 2223716, 2239952 |
| Penicillin G | 2220261, 2220288, 2220296 |
| Cefotaxime | 2434091, 2434105 |
| Clarithromycin | 2403196, 2274744, 2274752, 2413345, 2146908, 2244641, 1984853, 2126710, 2324482, 2324490, 2408988, 2408996  2442469, 2442485, 2466120, 2466139, 2471388, 2471396, 2247573, 2247574, 2346532, 2266539, 2266547, 2361426  2361434, 2390442, 2390450 |
| Erythromycin | 873454, 682268, 682276, 2326663, 1912755, 2225271 |
| IV Ceftriaxone | 2409968, 2499711, 2499738, 2287633, 2287668, 2250276, 2250292, 2292262, 2292270, 2292289, 2292297, 2325594  2325616, 2325624, 2325632 |

**S6 Table. Clinical Symptom Variables and Codes**

| Clinical Characteristics | Symptom | ICD-10 Codes | ICD-9 Codes |
| --- | --- | --- | --- |
| Symptoms related to Lyme Disease | Rash | R21* | 07821 |
|  | Fever | R50.8, R50.9 | 07806 |
|  | Fatigue | R53* | 78079 |
|  | Chills | R50.8, R68.8 | 07999 |
|  | Myalgia | M79.1 | 78096, 07291 |
|  | Headache | R51* | 07840 |
|  | Pain in joint | M25.5* | 71949 |
|  | Cervicalgia | M54.2 | 07231 |
|  | Disturbance of skin sensation | R20* | 07820 |
|  | Radiculopathy | M54.1 | 07244 |
| Musculoskeletal manifestations | Arthritis due to LD | M01.2* | 71100-9, 71190-9 |
|  | Arthralgia | M25.5* | 71140-9, 71180-9 |
|  | Joint inflammation | M01.3*, M01.8* | 8070-9 |
| Nervous system manifestations | Lymphocytic meningitis | G00.8, G00.9, G01*, G02.8*, G03* | 03209, 03207  03218, 03220 |
|  | Cranial neuritis | M79.28, M79.29, G52*, G53.1*, G53.2* | 07292, 03520, 03526, 03529 |
|  | Radiculoneuropathy | M54.1* | 07244 |
|  | Encephalomyelitis | G04*, G05* | 03234-5 |
|  | Bell’s palsy | G51.0 | 03510 |
| Cardiovascular manifestations | Atriventricular conduction defects - complete heart block, third degree heart block, high-grade atrioventricular block | I44.1, I44.2, I44.3 | 42612, 04260, 42610 |
|  | Myocarditis | I51.4, I40*, I41.0*, I41.2* | 04290, 42292, 04220 |
| Ocular manifestations | Conjunctivitis | H10.0, H10.1, H10.2, H10.3, H10.8, H10.9 | 37200, 37203, 37205, 37239 |
|  | Keratitis | H16.3 | 37050 |
|  | Uveitis | H20.0, H22.0 | 36400, 36403 |
|  | Papillitis | H46 | 37730 |
|  | Episcleritis | H15.1, H19.0 | 37900, 37909 |
| Persistent symptoms | Fatigue | R53* | 78079 |
|  | Headache | R51* | 07840 |
|  | Stiff neck or neck pain | M54.2 | 07236 |
|  | Arthralgia | M25.5* | 71949 |
|  | Myalgia | M79.1 | 07291, 78096 |
|  | Radiculopathy | M54.1* | 07244 |
|  | Problems with cognition or memory | F04*, F05.8, F05.9, F06.7, R41* | 02940, 29389, 02930, 03101, 02989 |

* All sub-codes under this code are included.

**S7 Table. Clinical Staging Code Algorithm for Early Localized Disease**

|  | | | | | | | |
| --- | --- | --- | --- | --- | --- | --- | --- |
| For a patient to be assigned as EARLY LOCALIZED STAGE, the following criteria must be met: | | | | | | | |
| Criteria: Mutual Exclusivity Component | | | | | | | |
| Patients must not be assigned as LATE DISSEMINATE STAGE | | | | | | | |
| Patients must not be assigned as EARLY DISSEMINATE STAGE | | | | | | | |
| AND | | | | | | | |
| Criteria: Symptom & Time Component | | | | | | | |
| Patients must meet at least ONE of the the following sub-criteria ±90 days from index date: | | | | | | | |
| OR | Subcriteria 2a: Patients must have the following combination of codes on the same hospitalization abstract at least **ONCE** in the follow-up period: | | | | | | |
|  | **Conditions** | **ICD-10-CA codes** | **ICD-9-CM codes** | **AND, any of:** | **Conditions** | **ICD-10-CA codes** | **ICD-9-CM codes** |
|  | LD | A69.2 | 08881 |  | Erythema | L54*, L50*, L53.8, L53.9 | 06950, 07080, 69589, 06959 |
|  |  |  |  |  | Swelling, localized | R22* | 07822 |
|  |  |  |  |  | Cellulitis | L03* | 68100 |
|  |  |  |  |  | Rash | R21* | 07821 |
|  |  |  |  |  | Fatigue, Weak, Lethargy, Malaise | R53* | 78079 |
|  |  |  |  |  | Headache | R51* | 07840 |
|  |  |  |  |  | Myalgia | M79.1 | 07291 |
|  |  |  |  |  | Chills | R50.8, R68.8 | 07806, 07999 |
|  |  |  |  |  | Back pain | M54.5, M54.0* | 07242, 07236 |
|  |  |  |  |  | Body, generalized aches | R52* | 78096 |
|  |  |  |  |  | Fever | R50.8, R50.9 | 07806 |
|  | Subcriteria 2b: Patients must have the following code in their follow-up period: | | | | | | |
|  | ICD-10-CA: A26.0/ ICD-9-CM: 00271 | *Erythema migrans* | | | | |  |

**S8 Table.** **Clinical Staging Code Algorithm Criteria for Early Disseminated Disease**

| For a patient to be assigned as EARLY DISSEMINATED STAGE, the following criteria must be met: | | |
| --- | --- | --- |
| Criteria: Mutual Exclusivity Component | | |
| Patients must not be assigned as LATE DISSEMINATED STAGE | | |
| AND | | |
| Criteria: Symptom & Time Component | | |
| Patients must have at least TWO* of any of the following conditions occurring within ±90 days from index date: | | |
| Conditions | **ICD-10-CA codes** | **ICD-9-CM codes** |
| Bell's palsy/other cranial neuritis | G51.0, M79.28, M79.29, G52*, G53.1*, G53.2* | 03510, 07292, 07292, 03520, 03529, 03526 |
| Neck pain/neck, stiff | M54.2 | 07231 |
| Paresthesia | R20.2 | 07820 |
| Cognitive impairment/mood disturbance | F07.9, F06.3, F38*, F39*, F60.3* | 03109, 29383, 29660, 29690, 03013 |
| Visual symptoms | R44.1 | 36816 |
| Auditory symptoms | R44.0 | 07801 |
| Dizziness | R42* | 07804 |
| Lymphocytic meningitis/encephalitis/encephalomyelitis | A87.2, G04*, G05* | 00490, 03235, 03234 |
| Radiculoneuropathy | M54.1* | 07244 |
| Palpitations/arrhythmia | R00.2, I49* | 07851, 42741 |
| Chest pain | R07.3, R07.4, R07.1, I20.9 | 78659, 78650, 78652, 04139 |
| A-V heart block [second or third degree] | I44.1, I44.2, I44.3 | 42612, 04260, 42610 |

**S9 Table.** **Clinical Staging Code Algorithm Criteria for Late Disseminated Disease**

| Criteria: Symptom & Time Component | | |
| --- | --- | --- |
| Patients must have at least ONE of any of the following conditions occurring within ±90 days from index date: | | |
| Conditions | **ICD-10-CA codes** | **ICD-9-CM codes** |
| Arthralgia | M25.5* | 71949 |
| Joint inflammation | M01.2*, M01.3*, M01.8*, M02.8*, M03.2*, M03.6*, M13*, M14.8*, M00.8, M00.9 | 71189, 71149, 71189, 71689, 71149, 71182, 71650, 07138, 71109, 71109 |

S10 Table. Disease Distribution of 3-Digit LD Codes (April 1, 2015 – December 31, 2021)

| Distribution of 088.xx codes during April 1, 2015 – December 31, 2021 | | |
| --- | --- | --- |
| ICD-9 CM Code | **Definition** | **Frequency** |
| 088.81 | Lyme Disease | 4,136 (95.6%) |
| 088.0 | Bartonellosis | 83 (1.9%) |
| 088.82 | Babesiosis | 53 (1.2%) |
| 088.9 | Arthropod-borne disease, unspecified | 47 (1.1%) |
| 088.89 | Other specified arthropod-borne diseases, other | 9 (0.2%) |
