# Supplementary material for: Use of administrative data for evaluating trends in medically-attended Lyme disease, Manitoba, Canada, 2010–2021
Source: PLoS One. 2026 Apr 13;21(4):e0342260. doi: 10.1371/journal.pone.0342260 (PMC13075671; doi:10.1371/journal.pone.0342260)
Supplement: S3 File — (DOCX) [file pone.0342260.s003.docx]

**Appendix: List of Databases**

| **Database** | **Years** | **Data Fields/Variables** | **Rationale** |
| --- | --- | --- | --- |
| Drug Program Information Network (DPIN) | January 1, 2009 – December 31, 2022 | - Scrambled PHIN - Date Prescription Provided - Days Supplied - DIN - Dosage (Strength) - ATC Code - Drug Cost Claimed - Drug Cost Paid - Product Description - Product Name | Identify concomitant medications at baseline.    Ascertain outcome of antibiotic prescriptions. |
| CIHI-DAD Hospital Discharge Abstracts (Manitoba Health) | January 1, 2009 – December 31, 2022 | - Scrambled PHIN - Admission Date - Separation Date - Diagnosis Codes - Intervention Codes - Intervention Dates - Length of Stay - Resource Intensity Weights | Identify comorbid conditions as covariates.  Identify Lyme Disease symptoms, diagnoses and reinfections.  Ascertain outcomes of costs, hospitalizations, surgical procedures and outpatient physician visits. |
| Medical Services Claims (MHSC) | January 1, 2009 – December 31, 2022 | - Scrambled PHIN - Date of Service - Tariff Code - Diagnosis Code - Specialty Sub-bloc - Number of Services Provided - Physician type / specialty - Fee charged | Identify comorbid conditions as covariates.  Identify Lyme Disease symptoms. diagnoses and reinfections.  Ascertain outcomes of costs, surgical procedures and outpatient physician visits. |
| Manitoba Health Insurance Registry | January 1, 2009 – December 31, 2022 | - Scrambled PHIN - Sex - Date of Birth - Coverage Dates (start/end) - Reason for Coverage Cancellation - Postal Code - Municipality Code | Determination of gender, age, and postal code (area of residence, income quintile) for baseline demographics.    Determination patient coverage for censoring.  Identify all-cause mortality as an outcome. |
| National Ambulatory Care Reporting System (NACRS) | January 1, 2009 – December 31, 2022 | - Scrambled PHIN - Date of ED Visit - Reason for ED Visit - Diagnosis Codes | Identify Lyme Disease diagnoses, symptoms, reinfections, and Emergency Department visits for Lyme Disease. |
| Cadham Provincial Laboratory Information Management System (LIMS) | January 1, 2010 – December 31, 2022 | - Scrambled PHIN - Specimen received - Date of specimen taken - Specimen source - Test Result - Code for serology - Parasite and microbiology result - Virus code - Code for organism | Identify serology tests for Lyme Disease as an outcome. |
| Long Term Care Utilization | January 1, 2009 – December 31, 2022 | - Scrambled PHIN - Date of LTC Residency | Identify long term care at baseline and as an outcome. |
| Home Care Utilization | January 1, 2009 – December 31, 2022 | - Scrambled PHIN - Date of Home Care Residency | Identify home care at baseline and as an outcome. |
| National Reporting System | January 1, 2009 – December 31, 2022 | - Scrambled PHIN - Date of Rehabilitation Services | Identify rehabilitation services at baseline and as an outcome. |
| Emergency Department Information System (EDIS) | January 1, 2009 – December 31, 2022 | - Scrambled PHIN - Triage information - CTAS Score - Length of stay - Transfer and discharge information | Identify Lyme Disease symptoms. diagnoses and reinfections. |
| MSSP Home Care Data | January 1, 2009 – December 31, 2022 | - Scrambled PHIN - Date of Home Care Residency | Identify home care at baseline and as an outcome. |
| MDS Home Care Data | January 1, 2009 – December 31, 2022 | - Scrambled PHIN - Date of Home Care Residency | Identify home care at baseline and as an outcome. |
| Procura Home Care Data | January 1, 2009 – December 31, 2022 | - Scrambled PHIN - Date of Home Care Residency | Identify home care at baseline and as an outcome. |
| MDS Long Term Care Assessment | January 1, 2009 – December 31, 2022 | - Scrambled PHIN - Admission date to Long term care - Discharge date to long term care | Identify long term care at baseline and as an outcome. |
