# Supplementary material for: Use of administrative data for evaluating trends in medically-attended Lyme disease, Manitoba, Canada, 2010–2021
Source: PLoS One. 2026 Apr 13;21(4):e0342260. doi: 10.1371/journal.pone.0342260 (PMC13075671; doi:10.1371/journal.pone.0342260)
Supplement: Attachment — Submitted filename: Response to Reviewers (PONE-D-25-22049).docx [file pone.0342260.s005.docx]

Maria Major, PhD

Medical Affairs Scientist

Pfizer Canada

October 15, 2025

Stephen M. Rich, MS, PhD
Academic Editor
PLOS ONE

Dear Mr. Rich,

Thank you for consideration and review of our manuscript: “Use of Administrative Data for Evaluating Trends in Medically-Attended Lyme disease, Manitoba, Canada, 2010-2021” (PONE-D-25-22049). Please accept submission of the revised manuscript (marked up and unmarked versions), which addresses the points raised during the review.

**Below are updates made related to the journal requirements:**

1. **Style Requirements:** I have amended the format of the authors’ affiliation and credentials, as well as the header font.
2. **‘Funding Information’ and ‘Financial Disclosure’ sections do not match**

Pfizer provided the sole funding source for this study. There was no grant number associated with this funding award, since I am an employee. This project was part of my PhD studies and was conducted as part of their funding of my studies.

3. **Updated financial disclosure statement:** The funders had no role in study design, data collection and analysis, decision to publish, or preparation of the manuscript.
4. **Funding and Competing Interests Statements:**
   a. **Updated Funding Statement:** The funder provided support in the form of salaries for authors but did not have any additional role in the study design, data collection and analysis, decision to publish, or preparation of the manuscript.
   b. **Updated Competing Interests Statement:**

Maria Major, Sarah J. Willis, Kate Halsby, James Stark, Ana Gabriela Grajales, Holly Yu, Frederick J.  Angulo, Chloe McDonald are employees of Pfizer, and as part of their compensation, may hold Pfizer stock.

Natalie Nightingale, Irene Wang, Calum Neish, Doneal Thomas, Saranya Nair, and Doneal Thomas are employees of IQVIA Solutions Canada Inc., which was a paid contractor to Pfizer in connection with the development of the study design and management.

Mark Loeb received compensation from Pfizer for his services as a member of the Lyme Disease Steering Committee.

The specific roles of these authors are articulated in the ‘author contributions’ section. This does not alter our adherence to PLOS ONE policies on sharing data and materials.

5. **Updated Data Availability Statement:** I have uploaded the full dataset from our study to the supporting materials (Supplement 2, S11 and S12 Tables).
6. **Abstract:** I have copied the revised abstract to the online submission.
7. **Supporting Information Captions:** have been added at the end of the manuscript.
8. **Reviewer comments included previously published work:** Kugeler et al. 2021 (Emerging Infectious Diseases 27(2)) was added to the references and mentioned in the introduction (lines 65-67).

**Below are updates made related to the journal requirements:**

**Note: Line number references below refer to the unmarked version of the manuscript.**

**Reviewer #1:**

- Personal Health Identification Numbers mentioned on line 93. Description of unique identifiers that prevent cases from being double counted added (lines 116-117). LD cases were not double-counted if they had subsequent LD-related healthcare visits.
- Full dataset was uploaded to the supporting information, which provides more detail on how many patients were identified by each algorithm (Supplement 2).
- Information regarding positive two-tier test results using CDC criteria for interpretation of western blots added (lines 110-111).
- Table 2 was reformatted to landscape orientation to fit widthwise on the page.
- Removed mention of boreal forests being unsuitable for tick habitats.
- Kugeler et al. 2021 (Emerging Infectious Diseases 27(2)) added (lines 65-67).
- CDC definition of Lyme disease incidence to define endemic areas was removed.

**Reviewer #2:**

- Inconsistencies referring to Ontario have been corrected.
- A combined study protocol was written for Manitoba and Ontario and was submitted for ethics review as such. To reduce confusion, I have removed the Waterloo ethics review and retained the University of Manitoba ethics review, which was the primary review for this study.
- The rationale for the algorithm precedence, was to capture the most clinically relevant outcome. This has been added to the manuscript (lines 121-122).
- Table 1 has been amended with the age groups condensed to 10-year groupings.
- Regional analysis was removed from Table 2. However, references to RHA and age group analysis remain in the methods section, as these results have now been uploaded as supporting information (Supplement 2; S12 Table).

I look forward to hearing your decision on the manuscript status. Please let me know if you need additional revisions.

Sincerely,

Maria Major
