# Supplementary material for: Use of administrative data for evaluating trends in medically-attended Lyme disease, Manitoba, Canada, 2010–2021
Source: PLoS One. 2026 Apr 13;21(4):e0342260. doi: 10.1371/journal.pone.0342260 (PMC13075671; doi:10.1371/journal.pone.0342260)
Supplement: Attachment — Submitted filename: Response to Reviewers (PONE-D-25-22049R1).docx [file pone.0342260.s006.docx]

Maria Major, PhD

Medical Affairs Scientist

Pfizer Canada

January 15, 2026.

Jorge Cervantes

Academic Editor
PLOS ONE

Re: Resubmission of manuscript “Use of Administrative Data for Evaluating Trends in Medically-Attended Lyme disease, Manitoba, Canada, 2010-2021” (PONE-D-25-22049R1)

Dear Dr. Cervantes,

Thank you for consideration and review of our manuscript. Please accept this revised manuscript (marked and unmarked versions), which address the points raised by reviewer 1, in item 6 of the review. All other items of the review appear to have no further questions or requested revisions.

**Item 6. Review Comments to the Author**

Reviewer #1: This manuscript is close to being ready for publication. My concerns regarding possible double counting of cases seems to have been mostly answered. However, some concerns about the inclusion of laboratory test data remain. Why would patients with positive test results not appear in the other databases, which identify clinical management? I cannot know – for this review by either the provincial laboratory director or a medical officer of Manitoba Health, Seniors & Long-term Care.

Minor points:
In the introduction I would recommend using Borrelia burgdorferi sensu stricto rather than sensu lato. Early localised LD can appear as a rash, which can take the form of a bullseye. I would maybe change ‘influenza type symptoms’ to something like non-specific malaise as there are no respiratory manifestations of Lyme disease.
In the methods it should be stated that the Discharge Abstract Database is a product of the Canadian Institute of Health Information (CIHI).

**Response from authors:**

**Regarding the double counting of cases:** The laboratory test data was cross-checked with the primary algorithms. Subjects with positive test data who previously met any of the algorithms 1 through 3 (defined in Supplement file 1, S2 Table), were excluded. This is evident in the complete patient flow outlined in Supplement file 2, S11 Table. The number of cases identified with positive serology was initially 520. Then 114 cases were excluded due to having been identified by one of the previous 3 algorithms. The final number of cases identified solely by positive serology was 406.

Why these 406 patients did not appear in the other algorithms cannot be answered by this study. One could speculate that they were possibly misdiagnosed or had not sought care during the early stages of disease and these represent disseminated cases treated by specialists, particularly since serologic testing is not indicated for diagnosis of early localised stage of Lyme disease. This could also reflect coding practices, where the test was ordered before a definitive clinical diagnosis was made.

I added a sentence to the discussion to clarify to possible explanation for why patients identified by were not picked up by the clinical algorithms (Lines 243-245). I also added a reference to support the statement (number 28).

**Other revisions made:**

The introduction was revised to include Borrelia burgdorferi sensu stricto (Line 43).

In the introduction, ‘influenza type symptoms’ was changed to “non-specific symptoms such as malaise” (Lines 47-48).

The methods were revised to “Discharge Abstract Database (DAD), which is a product of Canadian Institute of Health Information (CIHI)” (Lines 104-105).

**Please revise the funding statement**

- This work was supported and jointly funded by Valneva and Pfizer as part of their co-development of a Lyme disease vaccine.

Please let me know if there are other questions.

Sincerely,

Maria Major
